# Professional Pebbles

**Published:** 1853-09

**Authors:** A. Stroller


### PROFESSIONAL PEBBLES;

### *Picked up on the Sea-Shore of Medical Science.*

BY A. STROLLER, M. D.

It is often necessary and good treatment, to stimulate constitutionally and at the same time, deplete locally.....Bear in mind, that there is a certain period in all inflammations, when stimulants prove to be antiphlogistics.....Lesion of structure, by no means necessarily involves lesion of function; a cancerous stomach still digests.....In only one case can you apply blisters to the scalp itself, and that is, in coma, with cool skin.....In treating local inflammatory affections, in the *early* stages of fever, let your treatment lean decidedly toward depletion; but in treating precisely similar affections, in the late stages, carefully stimulate; for debility is now at hand, and stimulants do *now* what depletives or antiphlogistics did before.....A rule for the use of wine in fevers: where the patient's strength will no longer warrant the use of antiphlogistics, when the pulse falls, and the skin loses its morbid heat—then you may stimulate. The pulse however, may not have fallen, but become very rapid and small; no matter how quick it may be, if small; for the wine, if properly used, will bring it down. A slow feeble pulse will be quickened, and a rapid small one will be made slower, by the proper use of wine and stimulants.

Chronic diseases require chronic remedies.....“It is a curious fact,” Dr. Graves, of Dublin, says, “that every chronic bronchial derangement, is accompanied by intestinal flatulence;” whereas Stroller's experience goes to show that it is not a fact—what say you?....Opium is often given, for the purpose of checking inflammation of mucous tissues: in such cases, one test of its favorable effect is, that if it produces its ordinary narcotism, you will probably be disappointed—its anti-inflammatory effect is not obtained. It may therefore, in such cases, be confidently given to any necessary extent, as long as its good effects appear, and until narcotism is produced ....Never employ astringents in diarrhœa and dysentery, till the active inflammatory stage is past. As a general rule, in the cases alluded to, use first the gentler vegetable astringents, reserving the mineral astringents till a more chronic stage.

The signs of disease, derived from the eye, must be cautiously received; for aberrations of vision are often dependant, rather on the degree of susceptibility of the patient, than on the amount of disease. In all cases of obstinate vomiting, look to the brain.....There is properly no such thing as *serous* apoplexy; what seems so, is usually sanguineous, or serous effusion without apoplexy. When, in fever, patients perspire very freely, from the first, the cases are usually tedious.....Gaspar and Magendie produced typhus fever, in animals, by injection of putrid substances into the veins. In the delirium of fever, not depending on cerebral inflammation, you are not to be deterred from the use of wine, because the eyes are red and suffused, if the other indications are favorable; for long continued want of sleep will often produce that appearance; and you may often use it even when the skin is hot, provided the extremities are too cool.

It is generally proper to give opium in typhoid fevers, where severe nervous symptoms remain, *after* appropriate antiphlogistic treatment, or where they occur, unaccompanied by signs of inflammation of the brain.....Erroneous views are sometimes taken of the *prostration*, incident to disease. It may be primary, depending on the violence of the invasion of the disease, or secondary, depending on local lesion, or it may be really the actual debility, produced by the disease. In all these cases, the treatment must be different.....Hippocrates says, that in all serious disease, where no pain is complained of, the brain is usually wofully at fault.....In cases of protracted confinement, to the horizontal posture, we often suspect serious pulmonary disease, bronchitis, or pneumonia, but can get no auscultatory signs of it. Why? because the respiration is so very feeble and imperfect; the lungs are not expanded and rales are slight. But set the patient up in bed, induce large respirations, and serious signs at once show themselves..... Watch, in the course of fever, for pulmonary disease, sympathetic with visceral: you will usually find it, first, in the lower lobe of the left lung, near to the stomach.....Supine position, long continued, in fever, often produces congestion of the posterior lobes of the lungs— a serious result.... Dyspnœa, depending on inflammation, may be relieved by bleeding; but dyspnœa, depending on excessive pulmonary secretion, will be augmented by it.

Do not give turpentine for tympanites, when active inflammation exists.....Not unfrequently, in the course of fever, inflammation and ulceration of the intestinal glands, goes on, and passes through all its destructive forms, without pain. How shall we detect it? Either by tenderness on pressure, (which is not always a certain sign,) or by these following symptoms: Thirst without vomiting; preference for warm drinks; tympanites, and diarrhœa. These symptoms indicate latent lesion of the glands of the small intestines. In such cases, if much thirst, sedulously avoid stimulants.....In fevers, the early use of active purgatives, tends to produce tympanites.....Thirst often depends not on the dryness of fauces, but on the state of the nerves of the stomach; for it may be relieved by the injection of water into the stomach, without touching the throat.....Watch closely for the onset of cerebral disease, and anticipate its approach. An early sign of cerebral disturbance, will often be found in irregular or sighing respiration—fast, slow and sighing—if, of course, there be no pulmonary disease to cause it: such has been called 'cerebral respiration.'....The pupil of the eye is permanently contracted, during sleep.....The more complicated any disease may be with others, the more latent is any local lesion.....Andral says no constant connection exists between the state of the tongue, and that of the stomach; and Louis confirms this opinion. Regard the tongue, says Stokes, not as an indication of any local disorder, even of the alimentary canal, but as an index of the general condition of the whole system.....You cannot always remove sympathetic derangements by removing the cause; it may have gone on too long, and have produced a state of actual organic disease.

Lesions of the alimentary canal are very apt, especially in children, to subside, apparently, after a time, and reappear in the head or lungs. This metastasis, however, is more apparent than real; inflammation may begin in the new place, but does not on that account subside in the former.....Opposite conditions of the system are often accompanied by similar symptoms.....In inflammation of viscera, the severity of the pain, other things being equal, is usually in proportion to the superficiality of the local inflammation.....The specific effects of Hydrarg, are much more readily obtained by prefacing its employment by the use of remedies calculated to relieve the existing inflammation—or, in conjunction with such remedies.

If you give iodide of potassium when free acid exists in the stomach, combine it with liquor potassæ; else, the salt is decomposed, and the iodine set free, may become an irritant.....Never apply leeches to the eye-lids, or scrotum; dangerous ædema is apt to ensue..........In cases of dropsy from disease of the heart, examine the urine; it will almost always be found albuminous.

